# An Autopsy Case of a 5-Year-Old Child with Acute Pancreatitis Caused by Eosinophilic Granulomatosis with Polyangiitis-like Necrotizing Vasculitis

**DOI:** 10.1155/2019/9053747

**Published:** 2019-09-02

**Authors:** Haruna Yagi, Seishiro Takahashi, Tetsuo Kibe, Kenji Shirai, Isao Kosugi, Hideya Kawasaki, Shiori Meguro, Toshihide Iwashita, Hiroshi Ogawa

**Affiliations:** ^1^Department of Diagnostic Pathology, Seirei Mikatahara Hospital, 3453 Mikataharacho, Hamamatsu 433-8558, Japan; ^2^Department of Regenerative and Infectious Pathology, Hamamatsu University School of Medicine, 1-20-1 Handayama, Higashi-ku, Hamamatsu 431-3192, Japan; ^3^Department of Pediatrics, Seirei Mikatahara Hospital, 3453 Mikataharacho, Hamamatsu 433-8558, Japan

## Abstract

In children, acute pancreatitis has been reported in IgA vasculitis, Kawasaki disease, systemic lupus erythematosus-associated vasculitis, and juvenile dermatomyositis-associated vasculitis. However, its frequency in these vasculitides has been shown to be low. In other childhood-onset vasculitides, acute pancreatitis is seldom reported. The patient was a 5-year-old Japanese boy who suddenly presented with gastrointestinal (GI) bleeding. Therapy with antiulcer drugs successfully stopped bleeding, but subsequently, high fever, leukocytosis, and hypoxia appeared. He died 12 days after he presented with GI bleeding. An autopsy unexpectedly revealed that necrotizing vasculitis with marked eosinophilic and histiocytic infiltration of the pancreas led to acute pancreatitis, and gastric ulcer with eosinophilic infiltration was shown to be the origin of GI bleeding. In addition, eosinophilic infiltration was found in the small intestine, lungs, and bone marrow. Necrotizing vasculitis with eosinophilic and histiocytic infiltration of the pancreas, eosinophilic infiltration of the airway wall, and eosinophilic gastroenteritis with gastric ulcer were histologically confirmed, suggesting that the present case may be an early stage of eosinophilic granulomatosis with polyangiitis- (EGPA-) like vasculitis. To our knowledge, this might be the first reported case of EGPA-like vasculitis presenting with acute pancreatitis in a child.

## 1. Introduction

Vasculitides are rare and heterogeneous diseases that affect different organs with variable severity. According to the 2012 Revised International Chapel Hill Consensus Conference, vasculitides are classified into seven major categories: large vessel vasculitis, medium-vessel vasculitis, small-vessel vasculitis, variable-vessel vasculitis, single-organ vasculitis, vasculitis-associated systemic disease, and vasculitis associated with probable etiology [1]. Vasculitis can cause various symptoms depending on the organ involved. Gastroenteric lesions caused by vasculitis are relatively common, but pancreatic lesions are very rare [2, 3].

Acute pancreatitis caused by vasculitis is rare in children as well as in adults [2–4]. In children, acute pancreatitis has been reported in patients with IgA vasculitis, Kawasaki disease, systemic lupus erythematosus- (SLE-) associated vasculitis, and juvenile dermatomyositis- (JDM-) associated vasculitis [5–8]. In a study by Zhang et al., the occurrence of acute pancreatitis in IgA vasculitis was reported to be 0.4% (13/3212) [5]. A recent English study involving five cases of Kawasaki disease-associated pancreatitis indicated that pancreatitis could precede the onset of Kawasaki disease [9]. Acute pancreatitis occurred at the onset of childhood-onset SLE in 8% of cases [7]. Five JDM cases with pancreatitis have been reported [8]. Concerning other vasculitides, we could not find any report of acute pancreatitis caused by vasculitides in the English literature.

Here, we describe an autopsy case involving a 5-year-old child with acute pancreatitis caused by necrotizing vasculitis with marked eosinophilic and histiocytic infiltration, which we could not predict before his death. In this report, we discuss possible diagnoses of the present case among vasculitides.

## 2. Case Presentation

A 5-year-old boy suffered from cardiac arrest due to a water-related accident and experienced hypoxic-ischemic encephalopathy 4 years previously. Since then, his breathing had been supported by a mechanical ventilator in a care facility, and he had received clobazam for preventing epileptic seizures for the last one year. In his medical history, he seemed to have had no allergic disease.

Twelve days before his death, gastrointestinal (GI) bleeding of an obscure origin occurred, and he presented to our hospital. At the time of his admission to our hospital, his height and weight were 121.0 cm and 14.8 kg, body temperature was 36°C, blood pressure was 88/58 mmHg, and heart rate was 70–80 beats/min. The number of white blood cells (5240 cells/mm^3^) and eosinophils (430 cells/mm^3^) in the peripheral blood were within normal limits. Endoscopic examination was not performed. Use of a proton pump inhibitor resulted in the arrest of the GI bleeding. Thereafter, he was discharged from our hospital and returned to the care facility.

On the day of his death, because his body temperature increased to 39°C, he was readmitted to our hospital. His blood pressure was 91/51 mmHg; his heart rate was 170–180 beats/min, but heart sounds were normal. Rhonchi were heard in the chest wall, but wheezing was not heard. The abdomen was flat and soft. Cyanosis was noted in the extremities. No skin lesion was observed. Significant neurological deficits except for unconsciousness due to the hypoxic-ischemic encephalopathy were not identified.

Laboratory data demonstrated that the number of white blood cells increased (13,910 cells/mm^3^) but eosinophil count remained within normal limits (330 cells/mm^3^), and inflammatory markers and liver transaminases were elevated (C-reactive protein: 5.1 mg/dL; aspartate aminotransferase: 242 U/L; alanine aminotransferase: 227 U/L) (Table 1). Serum amylase and anti-neutrophil cytoplasmic antibody (ANCA) were not examined. Pulmonary infiltration was not seen on a chest radiograph, and no abnormal bowel gas pattern was detected in an abdominal radiograph. When we commenced treatment, cardiac arrest suddenly occurred, and he died soon after. An autopsy was performed to investigate the cause of the death and origin of the GI bleeding.

### 2.1. Pathology

**Figure 1:**
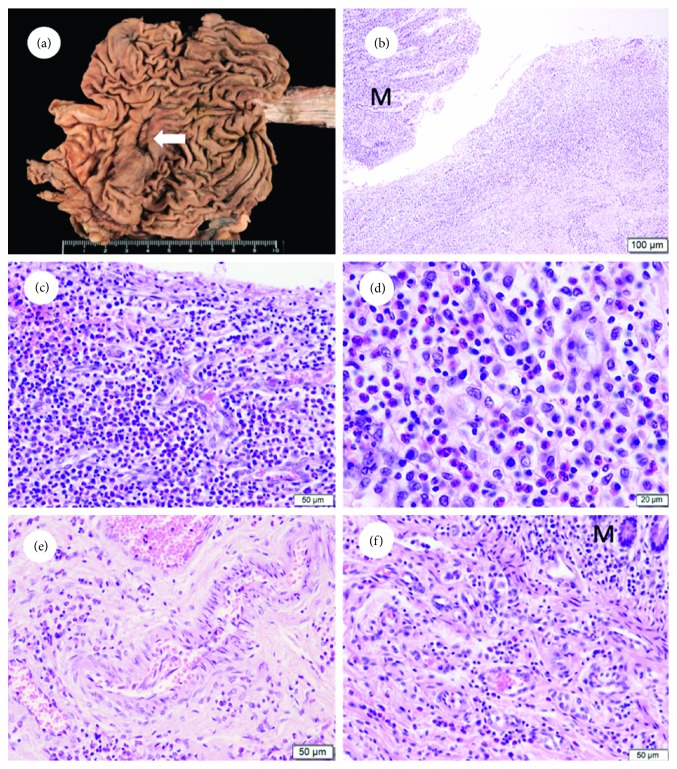
(a) Gross appearance of the gastric ulcer. In the gastric angulus, a 1.7 cm × 0.6 cm healing ulcer was observed. The arrow indicates the gastric ulcer. (b) Hematoxylin and eosin- (H&E-) stained figure of the gastric ulcer (×100). (c) H&E-stained figure of the granulation tissue of the healing stage of the gastric ulcer (×200). Eosinophilic infiltration is observed around capillaries without vasculitis. (d) Higher magnification (×400) of subfigure (c). H&E-stained figure of the submucosal layers of the stomach (×200) (e) and small intestine (×200) (f). Eosinophilic infiltration is observed without vasculitis. M: mucosal layer. Scale bars = 100 *μ*m (b), 50 *μ*m (c, e, and f), and 20 *μ*m (d).

In the gastric angulus, a 1.7 cm × 0.6 cm healing ulcer was observed (Figures 1(a) and 1(b)). Bleeding from the gastric ulcer was not observed. Mucosal erosion or ulceration was not identified in the small intestine, large intestine, or esophagus, indicating that the gastric ulcer was the source of GI bleeding, which occurred on the day 12 before his death. Histological examination of the stomach revealed that infiltration of numerous eosinophils was observed in the granulation tissue of the ulcer (Figures 1(c) and 1(d)). In addition, mild to moderate eosinophilic infiltration was observed in the mucosal and submucosal layers of the stomach (Figure 1(e)) and small intestine (Figure 1(f)). However, eosinophilic infiltration was not observed in the large intestine and esophagus. These results indicated that the gastric ulcer was due to eosinophilic gastroenteritis.

**Figure 2:**
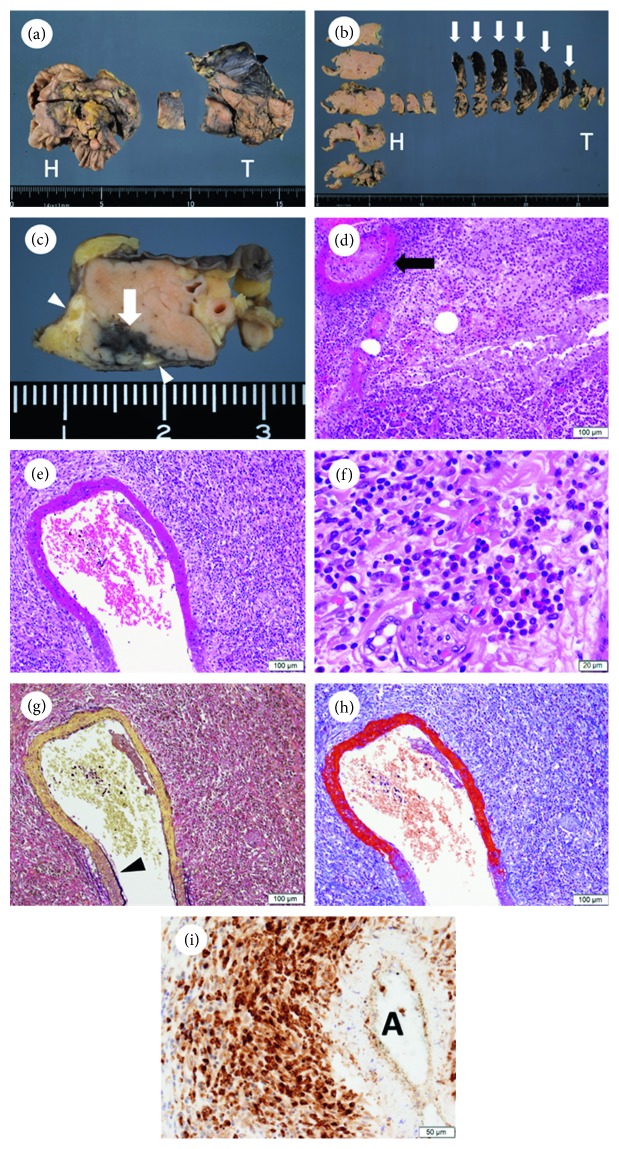
(a) Gross appearance of the pancreas. (b) Gross view of all sections of the pancreas. The arrows indicate hemorrhage spreading from the pancreatic body and tail to the retroperitoneal adipose tissue. (c) Gross view of a section of the pancreatic body. The arrow and arrowheads indicate the hemorrhage and fat necrosis, respectively. (d) Hematoxylin and eosin- (H&E-) stained figure of acute pancreatitis (×100). The arrow indicates necrotizing vasculitis of a small-sized artery. (e) H&E-stained figure of necrotizing vasculitis of a small-sized artery (×100). (f) Higher magnification (×400) of the adventitia of the small artery seen in subfigure (e). Infiltration by numerous eosinophilic cells is observed. (g) Elastica van Gieson-stained figure of a small-sized artery (×100). The arrowhead indicates the internal elastic lamina. The internal elastic lamina disappears in the upper part of the small-sized artery. (h) Masson's trichrome-stained figure of the small-sized artery (×100). Fibrinoid necrosis is accentuated by the red staining of Masson's trichrome stain. (i) Immunohistochemical staining indicates that the numerous infiltrating cells around the small-sized artery are CD163-positive histiocytes (×200). H and T in (subfigures a and b) indicate the head and tail of the pancreas, respectively. A: artery. Scale bars = 100 *μ*m (d, e, g, and h), 20 *μ*m (f), and 50 *μ*m (i).

Macroscopically, multiple hemorrhages and fat necrosis were observed in the pancreas (Figures 2(a)–2(c)). In particular, a considerable amount of hemorrhage had spread from the pancreatic body and tail to the retroperitoneal adipose tissue. Multifocal acute pancreatitis was histologically detected and shown to be associated with necrotizing vasculitis of small-sized vessels with numerous eosinophilic infiltration (Figures 2(d)–2(f)). Elastica van Gieson (EVG) staining revealed that the vessels had internal elastic lamina, demonstrating that they were small-sized arteries (Figure 2(g)). The fibrinoid necrosis in the vascular wall was accentuated by the red staining of Masson's trichrome stain (Figure 2(h)). As far as we investigated, no vasculitis was noted in the capillaries or veins of the pancreas. Immunohistochemical staining demonstrated that many CD163-positive histiocytes surrounded the injured small-sized arteries (Figure 2(i)). Vasculitis was observed in the pancreas alone.

**Figure 3:**
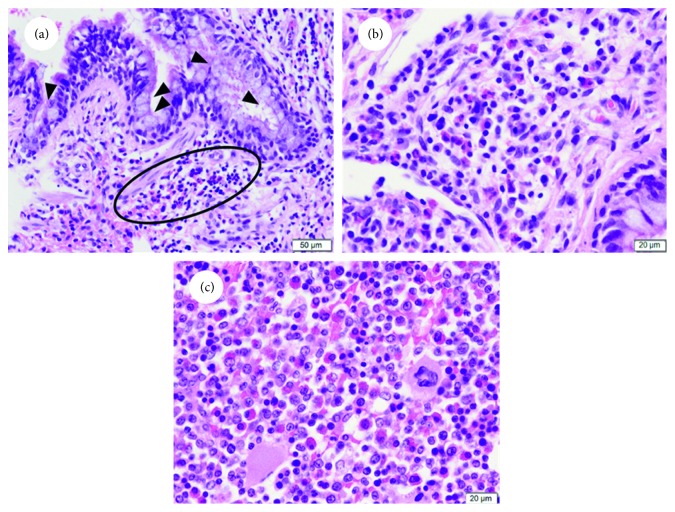
Histology of the lungs and bone marrow. (a) Hematoxylin and eosin- (H&E-) stained figure of the lungs (×200). Goblet cell metaplasia of the epithelium (arrowheads) and eosinophilic infiltration (ellipse) in the airway wall. (b) Higher magnification (×400) of subfigure (a). Infiltration of eosinophilic cells is observed. (c) H&E-stained figure of the bone marrow (×400). Marked increased eosinophil number is observed. Scale bars = 50 *μ*m (a) and 20 *μ*m (b and c).

In the lungs, eosinophilic and lymphocytic infiltration in the airway wall was widely observed. In addition, goblet cell metaplasia of the airway epithelium was observed (Figures 3(a) and 3(b)). The basement membrane beneath the airway epithelial layer was not thick, and neither hypertrophy nor hyperplasia occurred in smooth muscle cells around the airway epithelial layer. A bone marrow specimen demonstrated a hypercellular marrow with markedly increased eosinophil count, which made up around 20% of the total bone marrow cells (Figure 3(c)).

Other than in the pancreas, stomach, small intestine, lungs, and bone marrow, eosinophilic infiltration was not observed in the body organs. The heart and coronary arteries were macroscopically and histologically examined in detail, but no significant abnormality was observed. In the spleen, hemophagocytosis and neutrophilic infiltration were recognized. In the liver, centrilobular fatty degeneration with mild interface hepatitis was observed. No remarkable change was noted in the kidneys, bladder, adrenal gland, large intestine, esophagus, and gallbladder. Skin lesions, which are characteristic of IgA vasculitis, Kawasaki disease, SLE, and dermatomyositis, were not found. Histological examination of the central nervous system was not performed.

## 3. Discussion

The autopsy unexpectedly revealed that the patient suffered from acute pancreatitis before his death. No significant histological findings of severe shock or multiple organ dysfunction syndrome, which are usually seen in patients with severe acute pancreatitis, were detected. However, clinical manifestations and laboratory data on the day of his death met three out of four criteria of systemic inflammatory response syndrome (SIRS) (body temperature >38°C or <36°C, heart rate >90 beats/min, respiratory rate >20 or PaCO_2_ < 32 mmHg, and white blood cell count >12,000 cells/mm^3^ or <4000 cells/mm^3^, or >10% immature forms) [10], indicating that acute pancreatitis can lead to SIRS, which is associated with impaired cardiovascular function. Moreover, a considerable amount of bleeding caused by acute pancreatitis occurred in the pancreas and retroperitoneum, leading to hypovolemia. Therefore, we consider that the simultaneous occurrence of hypovolemia and SIRS may be the cause of the cardiac arrest in the patient.

Acute pancreatitis in the present case was due to necrotizing vasculitis of small-sized arteries with marked eosinophilic and histiocytic infiltration, which is also a histological hallmark of vasculitis in EGPA. The American College of Rheumatology (ACR) has proposed six criteria for EGPA (asthma, peripheral blood eosinophilia, mononeuropathy or polyneuropathy, nonfixed pulmonary infiltrates, paranasal sinus abnormalities, and extravascular eosinophilia), and the presence of four or more of these criteria is required for the diagnosis of EGPA [11]. In addition, Lanham et al. proposed three criteria for EGPA (asthma, peak peripheral blood eosinophil count >1500 cells/mm^3^, and systemic vasculitis involving two or more organs), and the presence of all criteria is required for the diagnosis of EGPA [12]. Regarding the present case, the presence of peripheral neuropathy was clinically unclear because the patient had been in a state of unconsciousness. The paranasal sinus was not clinically and histologically examined. Asthma was not clinically indicated, and pulmonary infiltrates were not detected in the chest radiograph although eosinophilic infiltration was histologically observed in the airway wall. The number of eosinophils did not significantly increase in the peripheral blood although it significantly increased in the bone marrow. Thus, the present case met only one criterion (extravascular eosinophilia) each out of the six criteria of ACR and the three criteria of Lanham. However, the autopsy revealed the existence of necrotizing vasculitis with marked eosinophilic and histiocytic infiltration. To date, only one EGPA case without eosinophilia like our case has been reported [13]. The present case may be the early stage of EGPA in which various symptoms have not yet appeared.

Although EGPA usually develops in adults, less than 100 childhood-onset cases have been reported in the English literature [14]. Eleftheriou et al. analyzed 13 cases of childhood-onset EGPA in the United Kingdom, and Fina et al. analyzed 14 cases in France. The most common presenting features showed pulmonary (Eleftheriou et al. vs. Fina et al.: 69% vs. 85%), skin (61% vs. 71%), digestive tract (46% vs. 64%), and cardiac (46% vs. 57%) involvement and a low rate of peripheral nerve involvement. Moreover, ANCA was negative in 10/13 vs. 10/14 patients [15, 16]. Zwerina et al. compared 33 childhood-onset EGPA with 205 adult-onset EGPA. Childhood-onset EGPA had a predominance of cardiopulmonary disease manifestations, a lower rate of peripheral nerve involvement, and higher mortality compared with adult-onset EGPA [17]. No significant difference was noted in the incidence of GI involvement between adult-onset and childhood-onset EGPA. As far as we know, acute pancreatitis in childhood-onset EGPA has not been reported.

The pathogenesis of EGPA remains unknown. EGPA is believed to be a result of complex interactions in which genetic and environmental factors cause inflammatory reactions [18, 19]. A previous report described the relationship between EGPA and the anticonvulsant drug carbamazepine [20]. In our case, to prevent epileptic seizures, the patient received clobazam, which has a different pharmacological effect compared to carbamazepine, for the last one year. Although no paper has noted the relationship between vasculitis and clobazam, we must always consider the relationship between anticonvulsant drugs and vasculitis.

Single-organ vasculitis (SOV) is one of the categories of vasculitis and a rare disease, which occurs in arteries or veins of any size in a single organ of an adult, and does not exhibit any feature that it is a limited expression of a systemic vasculitis [21]. In the digestive organs, approximately 80 cases of SOV in the GI tract, 24 cases of SOV in the gallbladder [22–26], and one case of SOV in the pancreas have been reported in English articles [27]. Similar to the present case, Carrascosa et al. reported a case of SOV in the gallbladder of an adult with necrotizing vasculitis of small- and medium-sized arteries and eosinophil-rich inflammation, but their case did not fulfill the criteria of ACR for EGPA [26]. In addition, Gonzalez-Gay et al. reported a case of SOV in the pancreas of an adult with polyarteritis nodosa-like arteritis in medium-sized peripancreatic arteries, but pancreatitis was not clinically or histologically observed [27]. The present case can also be diagnosed as childhood-onset SOV in the pancreas with marked eosinophilic and histiocytic infiltration.

In this case, the autopsy of a 5-year-old child unexpectedly revealed the existence of acute pancreatitis caused by EGPA-like necrotizing vasculitis. To the best of our knowledge, no similar report exists in the literature. The present case can be diagnosed as childhood-onset EGPA or childhood-onset SOV in the pancreas.

## Figures and Tables

**Table 1:** Laboratory data of the patient.

	On the day 12 before his death	On the day of his death		On the day of his death
White cell count (per mm^3^)	5240	13910	Aspartate aminotransferase (U/L)	242
Neutrophils (per mm^3^)	2170	7600	Alanine aminotransferase (U/L)	227
Eosinophils (per mm^3^)	430	330	Alkaline phosphatase (U/L)	2292
Basophils (per mm^3^)	30	70	Lactate dehydrogenase (U/L)	494
Monocytes (per mm^3^)	300	730	Creatine kinase (U/L)	60
Lymphocytes (per mm^3^)	2310	5180	Total protein (g/dL)	9.0
Red cell count (×10^4^ per mm^3^)	424	583	Albumin (g/dL)	3.8
Hemoglobin (g/dL)	12.4	16.9	BUN (mg/dL)	22
Hematocrit (%)	36.1	52.4	Creatinine (mg/dL)	0.46
Platelet count (×10^4^/*μ*L)	21.3	41.1	Total bilirubin (mg/dL)	0.5
			C-reactive protein (mg/dL)	5.1
